# Reducing human nitrogen use for food production

**DOI:** 10.1038/srep30104

**Published:** 2016-07-22

**Authors:** Junguo Liu, Kun Ma, Philippe Ciais, Stephen Polasky

**Affiliations:** 1 School of Environmental Science and Engineering, South University of Science and Technology of China, Shenzhen, 518055, China; 2 School of Nature Conservation, Beijing Forestry University, Qinghua East Road 35, Haidian District, 100083, Beijing, China; 3 Laboratoire des Sciences du Climat et de l’Environnement, CEA-CNRS-UVSQ, F-91191, Gif sur Yvette, France; 4 Department of Applied Economics, University of Minnesota, St. Paul, Minnesota, United States

## Abstract

Reactive nitrogen (N) is created in order to sustain food production, but only a small fraction of this N ends up being consumed as food, the rest being lost to the environment. We calculated that the total N input (TN) of global food production was 171 Tg N yr^−1^ in 2000. The production of animal products accounted for over 50% of the TN, against 17% for global calories production. Under current TN per unit of food production and assuming no change in agricultural practices and waste-to-food ratios, we estimate that an additional TN of 100 Tg N yr^−1^ will be needed by 2030 for a baseline scenario that would meet hunger alleviation targets for over 9 billion people. Increased animal production will have the largest impact on increasing TN, which calls for new food production systems with better N-recycling, such as cooperation between crop and livestock producing farms. Increased N-use efficiency, healthier diet and decreased food waste could mitigate this increase and even reduce TN in 2030 by 8% relative to the 2000 level. Achieving a worldwide reduction of TN is a major challenge that requires sustained actions to improve nitrogen management practices and reduce nitrogen losses into the environment.

Increased human production of reactive nitrogen (N) in fertilizers has increased crop yields^1^,^2^,^3^,^4^ to provide sufficient food for most of the global population. Yet, only a fraction of reactive N applied to croplands gets actually consumed in food, the rest being lost to the environment and negatively affecting soils, ecosystems, and climate via e.g. N_2_O and nitrogen aerosols emissions^5^,^6^,^7^. Agricultural production is currently responsible for the production of more reactive N than all terrestrial natural processes^8^. Crop production depends upon mineral fertilizers (Fig. S1), whose industrial production accounts for 53% of the total human creation of reactive N^9^, and it also involves biological fixation of N by cultivated leguminous plants. Extrapolation of the observed recent trends (Fig. S1) let us expect that by 2050, 70 to 100% more N fertilizer than in 2000 will be produced to meet the projected food demand by more than 9 billion people^10^,^11^. Future scenarios based on past trends coincide with assumptions on increased animal protein consumption, while population increase alone cannot explain the projected demand. If this scenario happens, it will imply massive losses of reactive N to the environment, with eutrophication, loss of biodiversity, air pollution via higher NO_x_ and NH_3_ emissions, water pollution, soil acidification, and emission of N_2_O^5^,^6^,^12^,^13^.

In this study, we calculated the total reactive N input (TN) associated with food production on a yearly basis. TN includes mineral and organic fertilizers applications, biological fixation, atmospheric deposition of nitrogen in croplands, and N recycling from crop residues, plus additional N uptake by crop plants from irrigation water and previously accumulated soil N pools. We first estimate the TN of human food production by combining N-use data for 20 crops^3^ with food consumption data^14^. Then we investigate future scenarios of agricultural and food waste management that could minimize TN while meeting the global food demand.

## Results

### Global TN of food production

**Figure 1:**
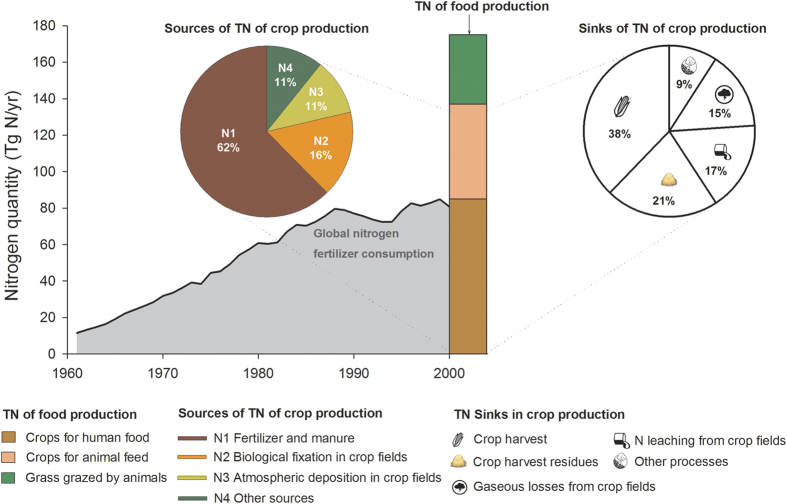
Total nitrogen input (TN) of food production. Left: Sources of reactive N involved in food production (animal and plant products). Right: Sinks of reactive N, showing the fate of this N. Less than 40% of the TN of crop production directly becomes human food.

In 2000, the global TN of food production is estimated to be 171 Tg N yr^−1^. This total is the sum of 137 Tg N yr^−1^ attributed to crop production^3^ (85 Tg N yr^−1^ for producing crops for human food, and 52 Tg N yr^−1^ for producing crops for animal feed) and 34 Tg N yr^−1^ to produce grass being grazed by animals (Fig. 1). A large amount of the reactive N included in this TN is lost to soils, aquatic systems and the atmosphere either before or after food consumption. We estimated the fraction of the TN lost to the environment during crop production before food consumption from models of nitrogen leaching^15^, gaseous emission rates (N_2_O, NO, and NH_3_)^16^,^17^, and soil erosion^18^. During crop production, leaching alone represents 17% of the TN of crop production, gaseous emissions 15%, and other processes 9% (these processes include reactive N remaining in soils, eroded material, and freshwaters, and transported to the coastal ocean by rivers). In sum, only 38% of the TN of crop production gets incorporated into harvested crop yields and 21% into crop residues, while the rest is lost without having been fixed by cultivated plants. Leaving crop residues in the field thus represents a recycling of N. Between yield harvest and final food consumption by humans, more than 20% of crop grains is further lost as food wastes. As a result of this cascade of N losses, out of the global TN of 171 Tg N yr^−1^ for food production, we estimated only 40 Tg N yr^−1^ (23%) ends up being consumed by humans as actual food (Fig. 1). The above estimate does not consider the food losses and waste of animal products because accurate data on these losses are not available. According to Gustavsson *et al*.^19^, the loss rates of meat products were all over 20%, while those of fish and seafood products were generally over 30% in different regions of the world. A conservative level of 20% for loss rates in the production and consumption of animal products gives a total N loss of 17.2 Tg N yr^−1^, and the percentage of N involved in animal products production ending up being consumed by humans of 13%. This result shows clearly that, today, losses dominate the TN of food production.

### Geographic distribution of TN

**Figure 2:**
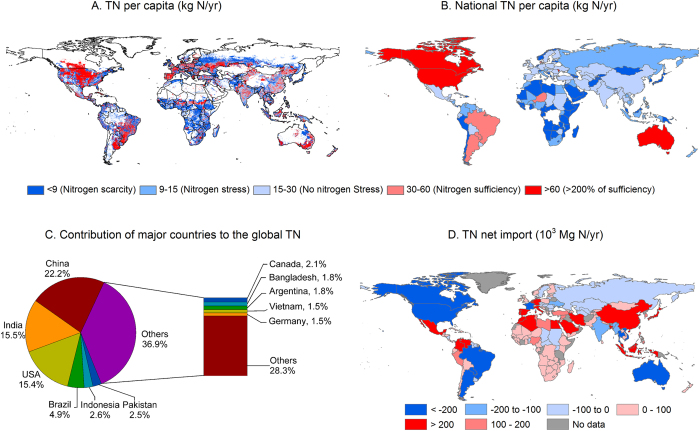
Geographic distribution of the TN of crop production and the TN associated with food trade. (**A**) Per capita TN at a 5-arc-minute resolution. (**B**) Mean per-capita TN at a national level. (**C**) Contribution of different countries to the global TN. (**D**) Net imports of TN associated with the crop trade. The top five food-exporting countries (the United States, Brazil, Canada, Australia, and Argentina) accounted for 60% of global TN exports. The top five food-importing countries (China, the Netherlands, South Korea, Japan, and Mexico) accounted for 40% of the global TN imports. [*Created with ArcGIS 9.3.1*].

**Figure 3:**
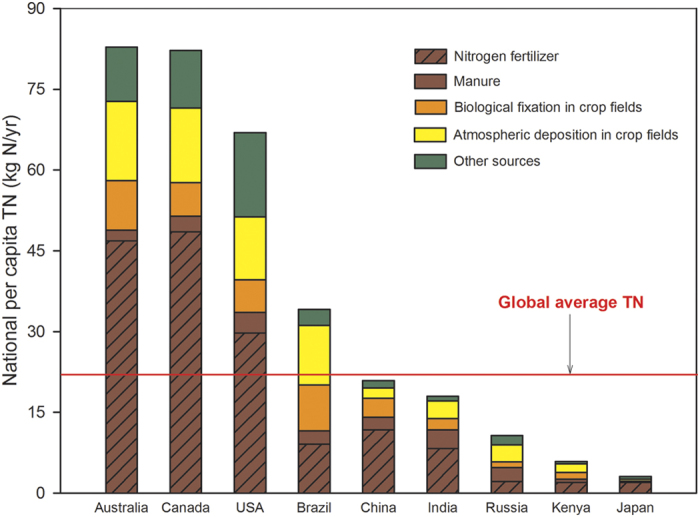
National TN of crop production on a per capita basis for several representative countries. It shows contrasts between counties (see text).

We then established the geographic distribution of the TN of crop production by using a spatially explicit nitrogen balance model^3^ (Fig. 2A–C). Figure 2A–C show that there are sharp contrasts in the per capita TN of crop production between countries (Fig. 3). Australia, Canada and the United States had the largest average per capita TN (83, 82, and 67 kg N yr^−1^ per person, respectively). These values are more than three times the world average of 22.3 kg N yr^−1^ per person and are typical of countries with nitrogen excess. Although China has the largest TN (27 Tg yr^−1^), because of its large population, China’s TN is only 21 kg N yr^−1^ per person, slightly lower than the global per-capita average. India’s per capita TN is 18 kg N yr^−1^ per person, 20% lower than the global per-capita average. By comparison, Kenya, a country representative of most African countries, has a low TN of 6 kg N yr^−1^ per person, 73% less than the per-capita world average.

Regions are grouped into five categories of N scarcity (per capita TN < 9 kg N yr^−1^), N stress (9 kg N yr^−1^ < per capita TN < 15 kg N yr^−1^), no nitrogen stress (15 kg N yr^−1^ < per capita TN < 30 kg N yr^−1^), N sufficiency (30 kg N yr^−1^ < per capita TN < 60 kg N yr^−1^), or nitrogen over 100% of sufficiency (per capita TN > 60 kg N yr^−1^) (Fig. 2A,B). N scarcity and N stress occur mainly in developing tropical countries, the Near East, and southern Russia, and affects 48% of the world’s population. By contrast, N sufficiency or excess^3^ affects 26% of the world’s population today. N sufficiency and excess for crop production occur mainly in countries where crop production is intensive, as shown in Fig. 2A. The above estimates were calculated by mapping the gridded information of N inputs with that of population, and this calculation neglects the role of global trade in allocating N among regions. Note that the global distribution of TN of crop production mapped in Fig. 2A does not take into account N flows within countries from crop production areas to consumers. In the USA for instance, nitrogen scarcity of the grid cells in West and East coast areas in Fig. 2A means a small TN of local food production, whereas this local scarcity is in the real world compensated by the import of N embedded in food produced in US agricultural production basins (e.g. Great Plains).

### Product TN

**Figure 4:**
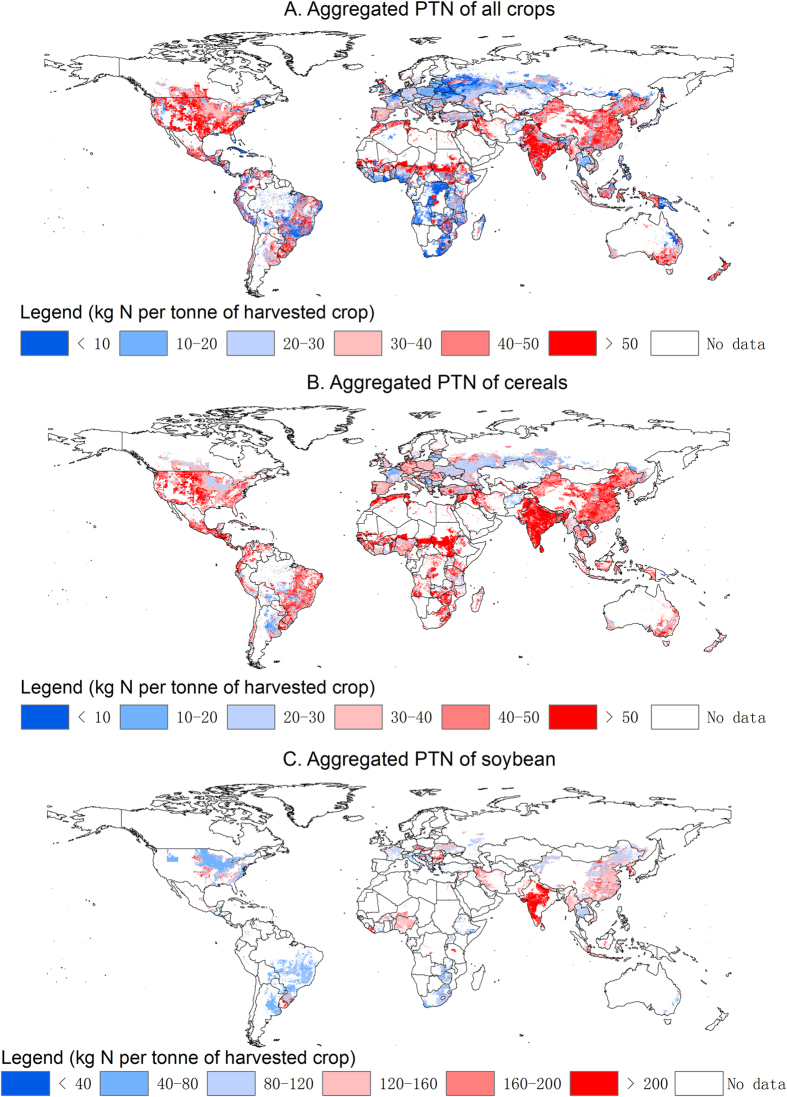
Spatial distribution of the product TN (PTN) of all crops combined, of cereals, and of soybean. This figure is generated at 5 min resolution using ArcGIS 9.3.1.

The nitrogen flows embedded in the trade of crop commodities depends on the amount of food traded and the TN of the food items. Product TN (PTN) is used to define the N consumed per unit of each specific crop food item, and the spatial distribution of the PTN is shown in Fig. 4. The world average PTN ranges from 40 kg N t^−1^ for cereals to 76 kg N t^−1^ for soybean, including for this plant, biological fixation and fertilization. PTN has an even large range when all products are considered, going from less than 5 kg N t^−1^ for sugar cane, sugar beet and cassava to over 100 kg N t^−1^ for the cultivation of groundnuts and beans. The PTN of crop also varies among different regions due to the difference in climatic and soil conditions, fertilization levels, and crop varieties. For all crops as a whole, the production-weighted average PTN in Africa (20.2 kg N t^−1^) appears to be 10% higher than that in Europe (18.3 kg N t^−1^). In Africa, cereal crops are currently grown without sufficient amounts of nitrogen and the yield is only about 40% of that of Europe^14^.

### TN embedded in trade

**Figure 5:**
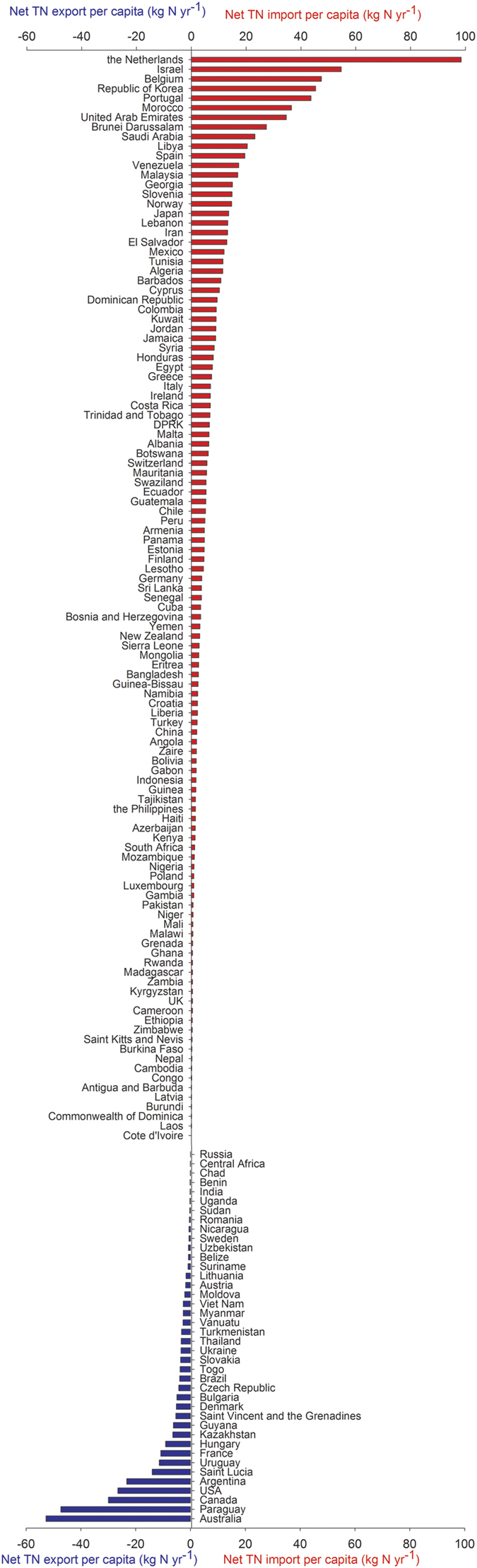
Per capita net import of TN at the national level. The red color shows net TN import, while the blue color shows net TN export on a per capita basis.

The nitrogen flows embedded in crop trade among different countries with various N inputs are presented in Fig. 2D. The TN embedded in crop trade ranges between 13 and 20 Tg N yr^−1^ (see calculation in *Methods Summary* and Table S1; national per capita net TN import in Fig. 5). Part of the excess TN in some developed countries is transferred to countries with N scarcity and stress via international trade of crop products. If we adopt a consumption based attribution perspective, almost 100% of the TN associated with imported food can be attributed to importing countries falling in the categories of N scarcity or stress (Table S1; Fig. 2D). Globally, consumers outsource more than 10% of their TN by importing food from outside their national boundaries. If animal products are considered, the patterns of TN embedded in trade are different. For example, the Netherlands was the biggest net TN importer for crop products on a per capita basis, but it is a net TN exporter for animal products. New Zealand imported a small amount of TN through crop trade, but it exported over 250 kg N yr^−1^ person^−1^ for animal products (Fig. 5).

### TN of vegetal compared to animal food production

**Figure 6:**
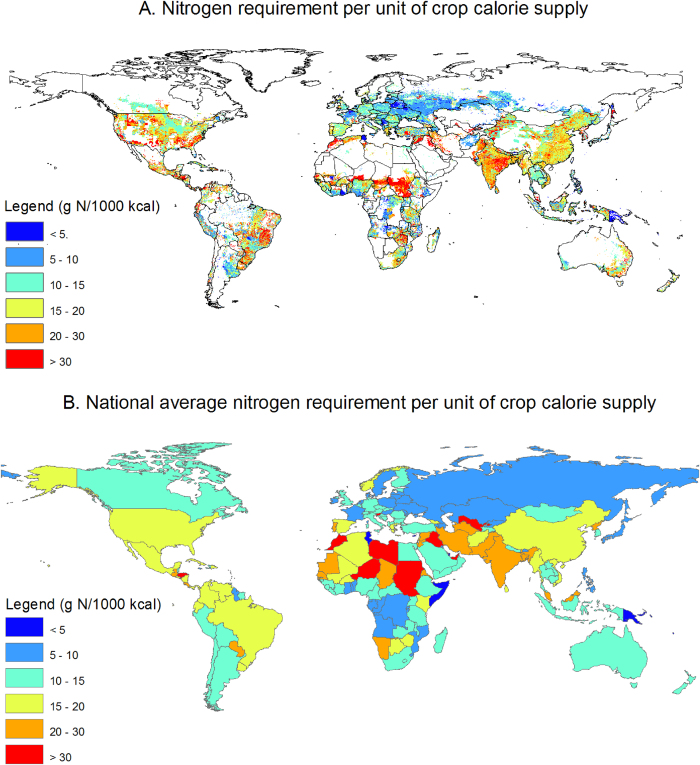
Spatial distribution in the nitrogen (N) required per unit of food calorie supply. The N required per unit of food calorie supply is the amount of nitrogen use (all types) required to produce each unit of caloric energy. The results show high spatial heterogeneity of the N requirement per unit of calorie supply among regions. [*Created with ArcGIS 9.3.1*].

We found that a global average of 16 g N is required to produce 1000 kcal of plant products (Table S2) by dividing the TN of crop production (weighted by crop-specific production data) by the dietary energy supply of all vegetal crop products^14^. This number varies largely among regions (Fig. 6). It should be noted that an important proportion^20^ (35%) of crop production has the purpose to feed animals rather than being converted directly into human food calories. A total of TN of 86 Tg N yr^−1^ is estimated for the total animal-feed production, 56% being from crop-derived feed and the rest from grazed grass in pasture^21^ (see *Methods Summary*). This result implies that the production of animal food accounts for more than 50% of the total TN, while it represents only 17% of the global food calorie production^14^. The resulting nitrogen requirement per unit of caloric production is of 84 g N per 1000 kcal for animal calories production (Table S2) compared with only 16 g N per 1000 kcal for vegetal calories production. The consumption of animal products thus requires six times more N than that of plant products for the same caloric production. Therefore, from a TN perspective, using cropland to produce animal feed, no matter how efficient, leads to much higher TN values. Nevertheless, animal protein is valuable for human diets, and requires at least plant protein with similar metabolic qualities as a replacement.

### Historical trends and future scenarios of TN

**Figure 7:**
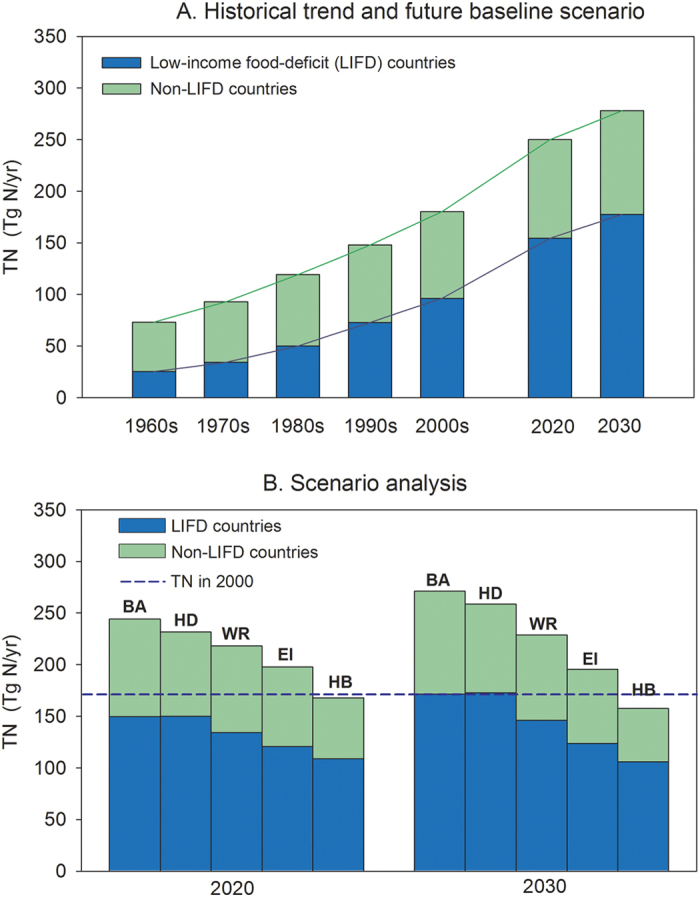
Historical trends for TN of global food production and the predicted TN to meet the hunger eradication target. (**A**) Historical trend for global food production and future baseline scenarios for 2020 and 2030. (**B**) Five future scenarios for TN required to meet the hunger eradication target. BA, *Baseline*; HD, *healthy diet*; WR, *waste reduction*; EI, *efficiency improvement*; HB, *combined*. A total of 175 countries were included in the analysis, including 69 low-income countries with a food deficit (see Supporting Information).

Figure 7 shows the historical trends and different future projections of TN in 175 countries, altogether accounting for 98% of the global TN. These data were calculated from the FAOSTAT agricultural statistics from 1961 to 2009, and projected to the future for 2020 and 2030 according to 4 different scenarios (more details on national distribution in Fig. S2). In our *baseline* scenario, a dietary shift is assumed which meets the hunger alleviation goal of the Millennium Development Goals (http://www.un.org/millenniumgoals/). This baseline scenario also assumes that by 2030, all people will have shifted to a “healthy” diet with a total per capita dietary calorie supply of 3000 kcal day^−1^, of which 20% coming from animal products^22^. The TN is assumed to linearly evolve. So, this baseline scenario already makes a rather optimistic hypothesis in terms of healthy diet preventing a huge increase of TN. In this scenario, the N requirement per unit of caloric production remains as from today’s technology, i.e. there is no change from current agricultural technology and cropland and livestock production practice with respect to nitrogen use in agriculture (see *Methods Summary*).

In the *baseline* scenario, TN is projected to rise from 171 Tg N yr^−1^ by year 2000 to 244 Tg N yr^−1^ (+43%) by 2020 (Fig. 7A). By 2030, a total increase of 100 Tg N yr^−1^ is projected above the 2000 level (+58%). In this scenario, low-income food-deficit countries (LIFD) have to increase their TN to feed their growing population (Fig. S2). In particular, the TN of LIFD countries is projected to double between 2000 and 2030, whereas the TN of non-LIFD countries increases by 25% only (Fig. 7A and Fig. S2).

Our *baseline* scenario, even with its optimistic hypothesis about diet, therefore implies a continuous and large increase of TN in the future as a consequence from meeting the global hunger alleviation target through an increase of food production in LIFD countries. This large increase of TN is likely to have very detrimental environmental and climatic impacts. We therefore investigated possible pathways to reduce TN while alleviating hunger by constructing four alternative scenarios. The *healthy diet* scenario assumes that, by 2030, all population with food security will follow the healthy diet, which was developed and recommended by Rockström *et al*.^22^; whereas the malnourished population will keep the same dietary energy intakes as in the *baseline* scenario. The *waste reduction* scenario assumes a reduction of food waste by 50% from 2000 to 2030, consistent with recent policy adopted by the European Union and a recommendation from the United Nations (http://www.europarl.europa.eu/sides/getDoc.do?type=REPORT&reference=A7-2011-0430&language=EN). The *efficiency improvement* scenario assumes that the PTN could be reduced everywhere to reach the current average of European countries by year 2030. This implies that less reactive N will be used to produce a unit of crop biomass (dry matter) so that the global nitrogen use efficiency of agriculture will be greatly improved. Finally, the *combined* scenario combines the storylines of the previous three scenarios, assumed to be additive.

The evolution of TN for each scenario (Fig. 7B) indicates that, in comparison with the *baseline* scenario, the projected TN increase could be significantly mitigated by the *waste reduction* (by 16%), *efficiency improvement* (by 28%), and *combined* scenarios (by 42%). The combination of food waste reduction and improved nitrogen-use efficiency thus represents the most effective strategy to prevent a large future growth of TN. The *healthy diet* scenario only slightly reduces TN compared with the baseline case, in both 2020 and 2030, which is not surprising because a healthy diet was already part of the baseline scenario storyline. The *healthy diet* scenario assumes that non-LIFD residents will reduce their food consumption, particularly of animal products, but the effect on TN is modest because these countries only represent 37% of the world population^14^. In the *combined* scenario, which adds all policies together, TN can be curved down and shows a slightly lower TN by 2020 (−2%) and a significantly lower TN by 2030 (−8%) compared to 2000. Joint implementation of *waste reduction*, *efficiency improvement* and *healthy diet* policies has the benefit of meeting hunger alleviation targets while keeping TN slightly below the 2000 level.

## Discussion

### Link with other studies

Our TN estimate of 171 Tg N yr^−1^ for both crop and livestock production in 2000 compares well with the 170 Tg N yr^−1^ from Smil^21^, but is smaller than the total amount of reactive N input to cropland and grassland of 249 Tg N yr^−1^ from Bouwman *et al*.^23^. The estimate of Bouwman *et al*.^23^ is higher than ours partly because it contains N inputs to grassland that are not used for food production, i.e. grasslands that are not grazed. Our TN estimate is larger than the 120 Tg N yr^−1^ of Galloway^24^ for the 1990 s, who only accounted for reactive N produced by the Haber-Bosch process and BNF from the cultivation of rice and legume.

Leach *et al*.^25^ estimated the nitrogen footprint from the total amount of reactive N released to the environment from food consumption, which assumes that all the N consumed (even the part contained in food) will be eventually released to the environment. Hence, their definition of nitrogen footprint is equivalent to the total amount of N used, which is similar to our TN definition. Yet, one difference is that Leach *et al*.^25^ excluded N lost in food waste at the retailer, food service, and end-consumer levels. Waste accounts for about 1/3 of the food used by end-consumers (e.g. for corn, see Leach *et al*.^25^). According to Leach *et al*.^25^, the per capita nitrogen footprint of the food sector is 30 kg N capita^−1^ yr^−1^ for the U.S. If food waste was included in the case of the US, nitrogen footprint would increase up to 45 kg N capita^−1^ yr^−1^, which would make the Leach *et al*. estimate very close to ours (47 kg N capita^−1^ yr^−1^). The Dutch per capita nitrogen footprint calculated by Leach *et al*.^14^ corrected for food waste is 33 kg N capita^−1^ yr^−1^ will however remain 28% lower than our estimate. Leach *et al*.^25^ also took N in wastewater into account, which was not considered in this study where the end-users of N are defined as humans consuming food.

We found that 38% of TN is incorporated into crop yields, close to the value of 33% that Galloway and Cowling^26^ defined as the ratio of N in harvested yield to input fertilizer. In our study, crop yields and residues together comprise 59% of the TN of crop production, which is close to the upper limit of the estimate of 56% from Smil^27^. We found that N leaching and gaseous losses account for 17% and 15% of total TN, respectively. These two percentages compare well with the 16% and 14% values from Bouwman *et al*.^28^.

Our result shows that losses dominate the TN of food production. Such a finding is in line and consistent with some existing literature. For example, the generic Tier 1 method of the IPCC guidelines^29^ indicated that about 30% (10–80%) of nitrogen inputs to soil can be lost as leached N, the emission factor for NO_x_-N and NH_3_-N can be about 10% (3–30%) for synthetic fertilizer, and even higher for organic fertilizers, some more as N_2_O-N depending on the soil and fertilizer type (generally less than 5%). The only well known report on food waste from The Food and Agricultural Organization of the United Nations (FAO) also indicated that around one-third of food produced for human consumption is lost or wasted globally^19^.

The future TN of food production ranges from 157 to 271 Tg N yr^−1^ in 2030 according to our scenarios. The upper limit is almost the same as the 270 Tg N yr^−1^ projected for 2050 by Galloway *et al*.^24^, and it is also close to the reactive N inputs to world agriculture of 268 Tg N yr^−1^ for 2030 from Bouwman *et al*.^23^ in their “adapting mosaic” scenario. Bouwman *et al*.^23^ projected future reactive nitrogen inputs to range 268–331 Tg N yr^−1^ for 2030 under the four Millennium Ecosystem Assessment scenarios. The estimates from Bouwman *et al*.^23^ are higher than this study and Galloway *et al*.^6^ probably because they include natural grasslands (see above). Our scenarios are based on coincident N-reduction measures in food consumption and production, whereas Bouwman *et al*.^23^ took only a *production* perspective (Table S3). Our baseline scenario projection for global TN is consistent with the independent estimate of reactive N inputs to the world agriculture from Bouwman *et al*.^23^. We project an additional TN of 100 Tg N yr^−1^ for crop production in 2030 in the baseline scenario, regrouping natural and anthropogenic nitrogen TN for the future. Currently, fertilizer accounted for 40% of the total TN. This fraction is expected to become higher in the future in the baseline scenario; hence, human will at least need to use additional nitrogen fertilizer input to food production of 40 Tg N yr^−1^. This amount is close to the 48 Tg N yr^−1^ from Leach *et al*.^25^. Erisman *et al*.^30^ also projected a similar increase of 50 Tg N yr^−1^ of nitrogen fertilizer from 2000 to 2030 in the worst case when fertilizer demand is the highest.

Both our study and Bodirsky *et al*.^31^ pointed out the key role of policy for improving nitrogen use efficiency and reducing food waste in reducing N impacts. Our results show that, in 2030, the global TN can be 28% lower in a scenario with increasing nitrogen use efficiency, in good agreement with Bodirsky *et al*.^31^. We projected that achieving a target of reducing food losses and waste can reduce global TN by 16% in 2030 in comparison to a baseline scenario, while Bodirsky *et al*.^31^ projected a reduction of 24% in 2050 when food waste reduction was combined with waste recycling.

### Novelty and limitations

In comparison with previous studies, we present the first attempt to integrate global nitrogen footprints from the perspective of food production, consumption and trade (Table 1). Most previous global N studies focused on nitrogen for food production (e.g. Smil^27^, Galloway *et al*.^24^ Bouwman *et al*.^2^, Liu *et al*.^3^; Bouwman *et al*.^23^), and few considered food consumption (e.g. Bodirsky *et al*.^31^) and nitrogen flows in trade (one available example i.e. Galloway *et al*.^6^). As for food consumption, Bodirsky *et al*.^31^ provided a coarse-scale assessment for 10 world regions, while we compiled data from 175 countries. As for food trade, Galloway^6^ assessed N in traded fertilizer, grain and meat products, while we assessed N embedded in the trade commodities and N used to produce these commodities in exporting countries, a term that is similar to virtual water flows^32^.

Another novelty is that this study quantitatively demonstrates the importance of reducing food losses and waste in mitigating TN. Reducing food waste is recommended as one of the key actions to produce more food with less pollution^33^. However, quantitative estimates for N mitigation potential of reduction of food losses and waste remain extremely rare, except for ref. 31. Our main result is that reducing food losses and waste could help significantly reduce nitrogen footprint.

There are several possible improvements of our methodology. First, We did not account for vegetables in the TN of crop production. Vegetables have the highest TN after meat due to the small fraction of harvested plant biomass, high N input rates especially in developing countries and high waste fraction. The share of N fertilizers to grow vegetables is however low (<1% in Germany, Austria, the Netherlands and Norway, 2.0% in France, 4.4% in Argentina, and 6.4% in China)^34^. There is a sharp increase in vegetable demand in China due to population growth, and increasing N inputs is one important way to enhance vegetable production. Hence, omission of vegetables will not lead to large systematic errors in TN of crop production.

Second, in reality, a reference “healthy but not wasteful” diet is in fact quite variable among different regions, depending on parameters like metabolic need, age, or simply human biomass. However, there is no information about country-specific healthy calorie intake requirements. Hence, in this study, we used a reference diet of 3000 kcal per capita per day following the value of Rockström *et al*.^22^. This reference diet is based on the production level required to eradicate hunger recommended by the World Health Organization (WHO) and FAO^35^,^36^, and it includes the average food losses and waste (in terms of calories 24%; Kummu *et al*.^37^). Several other authors also used such a level as an indicator for food security, e.g. Kummu *et al*.^38^ and Gerten *et al*.^39^. Nevertheless, access to country- even local-specific caloric values will produce more reliable results. We also acknowledge the fact that our estimates are based on the level of calorific values, while protein contents were not considered. This is a shortcoming of most available literature, which estimated natural resources requirements (e.g. water and land) mainly based on calorie intakes rather than protein intakes^22^,^39^. Future research should put more efforts to investigate the relations between human diets and protein intakes. Human diets that are lacking of meat generally require more high-protein plants (e.g. soybean), which in turn need more nitrogen to grow. For example, on average, it takes 34.1 g nitrogen to produce 1000 kcal of soybeans, which is 41% of that to produce the same amount of animal product.

### Policy implication

Our results highlight the central role of combining different policies in order to jointly achieve hunger alleviation and mitigate the increase of TN of food consumption. The key is joint implementation of policies aiming at *both* a reduction of food waste by 50% *and* a worldwide improvement of N efficiency to a level comparable to the one achieved by Western European countries. Reducing food waste within supply chains is a problem that has defied solution and that threatens agricultural and environmental sustainability^1^,^20^,^33^,^40^. There has been little progress towards the food loss reduction target established by FAO in 1945^40^. Many obstacles make this target difficult to tackle, including a lack of monitoring programs in most countries^40^ and a lack of governance and institutions^21^. Food waste reduction will need sustained efforts from governments as well as a transition in consumers’ behaviors and habits. For example, policy-makers in China are taking action to fight food waste through a “clean your plate” campaign by limiting government-sponsored banquets – serious enough that some Beijing restaurants have had a drop of annual business by 35%^41^.

Achieving a lower PTN is also an effective TN mitigation measure. In the *Our Nutrient World* report, an aspirational goal for a 20% relative improvement in full-chain nitrogen use efficiency by 2020 is promoted^33^. The ratio of applied nitrogen fertilizer to per unit of crop yield has decreased in Japan^42^, and the USA^43^,^44^. Despite this, according to a USDA-ERS report, the USDA recommended nitrogen management practices are not fully implemented by corn farmers in the US Midwest^45^. Reducing PTN and improving nitrogen use efficiency need a strict nitrogen policy from governments^42^,^43^, and improved nitrogen management practice by farmers^45^,^46^. However, regions differ in nitrogen challenges and policies: sub-Saharan Africa and Latin America have low nitrogen inputs in agricultural fields largely due to poor transport, market infrastructure and poor ratio of yield increase to fertilizer cost; many countries there emphasized the need for fertilizer subsidies to ensure food production^33^. Policies are needed not only to encourage more nitrogen use, but also to invest in transport, market, and targeted research, as well as to expand irrigation and improve other agronomic factors. In contrast, regions e.g. Europe and China have faced pollution problems largely because they use too much nitrogen. These regions have started to lower the fertilizer subsidies and emphasized the reduction of nutrient losses and pollution, but the effectiveness of nitrogen policies may be challenged by insufficient investment and high costs of wastewater treatment^33^,^45^. In these regions, there is a need to shift toward a new sustainable agriculture paradigm based on precision farming and sustainable soil management. For both the nitrogen-poor and nitrogen-rich regions, future efforts need to not only demonstrate best nitrogen practices in the field, but also emphasize long-term dialogue, education and training among all actors in the whole supply chain of nitrogen pathways^33^.

Food trade partly allows maintaining food security in regions where domestic production is insufficient. The consequence of economics driven trade choices on PTN are not taken into consideration, even though they also have environmental impacts and economic costs for producing countries. In some cases, importing a specific crop product comes with a lower PTN than if the same product was produced domestically (e.g. soybean imported from Brazil to China) but the opposite can also happen (e.g. rice imported from Thailand to Brazil). Globally, we calculated that trade allows a net reduction of TN of 5% as compared to a hypothetical world where all imported goods were produced domestically.

Mitigation actions, such as reducing food waste, and improving nitrogen use efficiency, will not only reduce local but also global environmental pollutions. The positive externalities from these active actions cannot be fully internalized under uncoordinated local policies^31^; hence, inter-governmental effort^33^ and global collaboration^31^ will be required to prevent a further growth TN that would cause high damage to the environment and economic costs.

## Methods Summary

### TN of crop production

The total nitrogen input (TN) of crop production is the sum of the inputs of all nitrogen types (*t*) associated with crop production for all crop products (*c*).


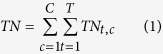


where *TN*_*t, c*_ is the TN of nitrogen type *t* (*t* = 1…*T*) for the production of crop type *c* (*c* = 1…*C*). Per capita TN is calculated by dividing TN by the population within the study area. The population data that we used were obtained from the Center for International Earth Science Information Network (CIESIN) at Columbia University (http://www.ciesin.org). The 2.5-arc-minute resolution population data for 2000 from CIESIN was converted into 5-arc-minute data by summing the values for adjacent 2.5-arc-minute cells, which were then used in this paper. Food production data are obtained from FAOSTAT^14^.

Six types of N use were accounted for in calculating TN for the production of each crop product: mineral nitrogen fertilizer (*TN*_*1, c*_); manure (*TN*_*2, c*_); wet and dry atmospheric deposition (*TN*_*3, c*_); biological nitrogen fixation (*TN*_*4, c*_); nitrogen inputs from water and from soil particles deposited by the wind or by flowing water (*TN*_*5, c*_), and nitrogen input from recycled crop residues (*TN*_*6, c*_). Estimates of the use of each nitrogen type were obtained from Liu *et al*.^3^ for different crops with a spatial resolution of 5 arc-minutes.

We considered 20 crops or crop groups: six cereal crops (wheat, rice, maize, barley, millet, and sorghum), three roots and tubers (potatoes, sweet potatoes, and cassava or yams), two pulse crops (dry beans and “other” pulses), two sugar crops (sugar cane and sugar beet), three fiber crops (coffee, cotton, and other fibers), three oil crops (soybeans, groundnuts, and other oil crops), and one fruit group (plantain and banana). These crops together accounted for almost 90% of the world’s total crop harvested area^3^.

### Product TN (PTN)

PTN is the amount of nitrogen used to produce one unit of crop product. For crop *c*, it can be calculated as the ratio of TN to crop production (*P*):


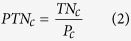


We calculated the average PTN for a group of crops (e.g., cereals) in each grid cell by dividing the total TN by the total production of all the crops in this group. We calculated the average PTN of a specific crop within a geographically delineated area as the ratio of the total TN to the total production of this crop within the geographic area.

The spatial distribution of crop production in 2000 was obtained from the Spatial Production Allocation Model (SPAM), which has a spatial resolution of 5 arc-minutes^47^,^48^. SPAM produced reliable spatially explicit crop production data, even for many developing countries (e.g., Sub-Saharan Africa and Brazil)^48^,^49^.

### N requirement per unit of caloric supply

The N requirement per unit of calorie supply is the amount of N (of all types) that is used to produce one unit of caloric energy. We calculated the aggregate N requirement per unit of calorie supply for a group of crops by dividing the total TN of all the crops by their total caloric energy. The TN of the consumption of a crop c is calculated by multiplying the PTN of this crop by the food intake from this crop. The total caloric energy intake from a crop c is calculated by multiplying the weight of food intake from crop type c by the caloric content of crop c. The data on the weight of food intake and caloric content of various crops are obtained from the Food and Agriculture Organization (FAO)^14^. We calculated the mean N requirement per unit of calorie supply of plant products (*n*_*p*_) with the equation below:


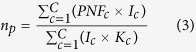


where *NR*_*c*_ is the N requirement per unit of calorie supply of crop type *c* (*c* = 1…*C*) (unit: g N/kcal), *I*_*c*_ is the weight of food intake from crop type *c* by an average person (unit: kg/person/year), and *K*_*c*_ is the caloric content of crop *c* (unit: kcal/kg).

We estimated that an average of 15.7 g of N is required to produce the equivalent of 1000 kcal of plant food. This value may represent a conservative estimate because we did not include vegetable oils in the calculation due to a lack of data. Vegetable oils account for 10.9% of the total dietary energy supply of plant products, and they usually have a higher TN and a lower calorie productivity per unit N when the entire supply chain is considered from oil crops to plant oils^14^.

A recent estimate suggests that 35% of crop production is used to provide animal feed, 62% for direct human food, and the remaining 3% for other purposes such as bioenergy or seed production and other industrial products^20^. This translates into 48 Tg N that is used to produce animal feed each year. Plants used to feed animals that are in turn used to produce animal products account for about 5/9 of the total TN of animal production, and the rest of the TN comes from grazing and aquatic foods^4^. Hence, the TN of animal products totals 86 Tg N yr^−1^. FAO reports that animal products provided 1.026 × 10^15^ kcal of dietary energy and 897.46 × 10^9^ kg animal products (the total weight of seven types, i.e. meat, offals, animal fats, eggs, milk, fish/seafood, and other aquatic products) in 2000. On average, it thus takes 83.9 g N to produce 1000 kcal of dietary energy from animal products, and 95.8 g N to produce each kg of animal products.

### Fate of N contained in the TN

In our analysis, we considered (1) N accumulated in the harvest crop (i.e., in the harvest yield); (2) N accumulated in residues of the harvested crop; (3) N leaching from crop fields into surface water or groundwater; (4) gaseous N losses from crop fields into the atmosphere as a result of volatilization and denitrification; and (5) N lost to soil erosion, and the resulting change in the soil N storage. Estimates of each flux were obtained from Liu *et al*.^3^, with a spatial resolution of 5 arc-minutes.

After a crop is harvested, part of the crop is lost through the processes of storage, processing, and transportation before it can reach humans for consumption. We also calculated N in such losses from the N that is accumulated in the crop yield. From a production perspective, we performed this calculation for all the crops based on the assumption that food loss rate (*l*) was constant for crops within the same crop category (e.g., cereals). TN lost through food losses or waste (TNL) was calculated by multiplying TN by *l*:


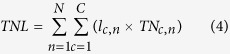


where *c* denotes the type of crops, and *n* denotes a country. Country- and crop-specific *l* values were obtained from Gustavsson *et al*.^19^ for five stages (harvest, postharvest handling and storage, processing and packaging, distribution, and consumption) for seven crop and meat groups (cereals, roots and tubers, oilseeds and pulses, fruits and vegetables, meat, fish and seafood, milk) in seven country groups. Thus far, Gustavsson *et al*.’s report is the most comprehensive source of global food waste statistics.

When our calculations were conducted from a production perspective, we did not consider food waste during the consumption process. But when we conducted such calculations from a consumption perspective, the food waste in the consumption process was taken into account. When predicting future TNL, we did not use detailed TN information for specific crops. Instead, we calculated TN based on dietary caloric intakes for plant and animal products. We used the *l* value of cereals to represent plant products, and that of meat to represent animal products. Cereals accounted for almost 60% of dietary energy intake from plant products, whereas meats accounted for 46% of dietary energy intake from animal products^14^.

### TN imports and exports

TN imports (TNI) and TN exports (TNE) were calculated based on Eqs (5) and (6), respectively. The TN net import (TNNI) equaled the difference between TNI and TNE. Country- and crop-specific trade data were obtained from FAO^1^.


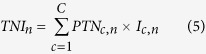



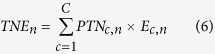



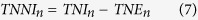


where *c* denotes the type of crops, *n* denotes a nation, *C* is the total number of crop types, *I*_*c,n*_ represents the units of crop *c* imported from country *n*, and *E*_*c,n*_ represents the export in crop *c* to country *n*.

### Historical and future TN of food consumption

We calculated the historical and future TN of food consumption (*TN′* ) considering plant and animal products for each scenario.


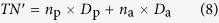


where *n*_p_ and *n*_a_ represent the N requirement per unit of calorie supply of plant products and animal products, respectively. *D*_p_ and *D*_a_ represent the dietary energy intakes of plant products and animal products per country, respectively. Historical values of *D*_p_ and *D*_a_ from the 175 countries that are monitored by FAO were obtained from FAO for the period from 1961 to 2009^14^. We estimated the future values based on the scenario analysis. We obtained data on historical and present national dietary energy supply per capita (both plant and animal calorie supply) from the FAO food balance sheets^14^. Population figures in each country from 1970 to 2030 were also obtained from FAO^14^. The future TN is compared with the result of the base year 2000.

### Quantifying the TN of food consumption to meet the hunger eradication targets (*baseline scenario*)

We analyzed the hunger eradication targets for the 175 FAO countries in two years: 2020 and 2030. For 2020, we assumed that the “hunger task” of the Millennium Development Goals (http://www.un.org/millenniumgoals/) would be fully achieved. This means that the proportion of undernourished people would decrease by half between 1990 and 2015. For 2030, we assumed that all people could achieve a “healthy but not wasteful” per capita calorie supply target (the “grand calorie target”) of 3000 kcal day^−1^, of which 20% comes from animal products (600 kcal day^−1^, the “animal calorie target”), following the value of Rockström *et al*.^22^.

Shifts of food consumption patterns and rising population are two key driving forces for food production. The global mean per capita calorie supply from animal products increased by more than 40% between 1970 and 2005^14^. This change was enormous for countries with the fastest growth of income and the fastest urbanization rate^14^. Hence, in our analysis, we considered the dietary calories from both animal products and plant products.

For 2020, we assumed that each undernourished person would eat 1700 kcal day^−1^ (an approximation of the basic human energy need), with the animal calorie supply equal to the present average for each country^14^. Adequately nourished people would at least reach and possibly exceed both the grand calorie target and the animal calorie target, and the caloric intakes should be no less than the level of 2000. For 2030, countries with a grand calorie supply in 2000 higher than the grand calorie target will remain at that level. For countries that have not achieved the grand calorie target, we assumed that they would reach this target in 2030 and that the calorie supply from animal products would be no less than the animal calorie target or the level of 2000.

The proportion of undernourished people in 1990 was based on available data for each country for the period from 1990 to 1992^14^. The nitrogen requirements to produce each unit of dietary calorie of plant or animal products were calculated in this study, as described above.

We aggregated the results of this analysis for 69 low-income food-deficit (LIFD) countries and 106 non-LIFD countries from the FAO list of 175 countries, for which calorie supply data are available. These LIFD and non-LIFD countries accounted for 63% and 36% of the world population, respectively, and they accounted for about 98% of total global TN. The lists of LIFD and non-LIFD countries are shown in the Supplementary Information.

### Scenarios for potential reduction of TN

In addition to the *baseline* scenario, we also set up four scenarios to study the potential for the reduction of TN:

#### S1 Diet-shift scenario

In this scenario, we assumed that all the population with food security would have the balanced diet (i.e., per capita consumption of 3000 kcal day^−1^ with 20% from animal products). For the malnourished population, we assumed that their dietary energy intakes would remain the same as in the *baseline* scenario (i.e., by 2030, the malnourished population also has per capita consumption of 3000 kcal day^−1^ with 20% from animal products). Such a consumption pattern means that currently affluent people would have to reduce their consumption, and particularly animal product consumption.

#### S2 Waste-reduction scenario

An international document disclosed to the European Parliament and the United Nations contains a commitment to the global reduction of food waste by at least 50% by 2025 in comparison with 1990 levels and also suggests that the reduction of food waste should be a new UN Millennium Development Goal (http://www.europarl.europa.eu/sides/getDoc.do?type=REPORT&reference=A7-2011-0430&language=EN). We used this commitment in our scenario, and assumed that progress would be made at a roughly equal rate each year. According to this commitment, food waste should be reduced by 50% in 2030 in comparison with 2000 levels. In S2, we assumed that this reduction task could be fully achieved in each country. The food loss rates in 2000 were based on an FAO report^19^ that summarized the rates for seven commodities for five phases of the supply chain in eight global regions. In this estimate, the food loss rates of the LIFD countries for plant and animal products were calculated by weighting the total food waste in all five supply chains for cereals, roots and tubers, oilseed and pulses, fruits and vegetables, and for meat, fish and seafood, and milk estimated by FAO based on the total food caloric supply of each food category in sub-Saharan Africa, North Africa, West and Central Asia, South and Southeast Asia, and Latin America from the 2012 FAOSTAT data^1^. Similarly, the food waste rates of the non-LIFD countries were calculated using weighted averages in Europe, North America, Oceania and industrialized Asia (Japan, China, and South Korea). Our calculations showed that food waste rates from “field to fork” reached 30.8% for plant products and 23.9% for animal products in LIFD countries, versus 38.7% for plant products and 22.6% for animal products in non-LIFD countries.

#### S3 Efficiency improvement scenario

PTN varies significantly among countries and continents. For example, among all the continents, Europe had the lowest PTN for cereals, with a value 30% lower than the world average. Here, we assumed that PTN in all countries would be reduced to the European level by 2030 at a linear rate over time. This assumes that the efficiency of N use will improve and that the N requirements per unit of calorie supply will consequently decrease.

#### S4 combined scenario

This is a combination of all the measures taken in scenarios S1, S2, and S3.

Uncertainties in the scenario results are analyzed by adjusting the major parameters (the “grand calorie target” of 3000 kcal day^−1^, the “animal calorie target” of 600 kcal day^−1^, the food waste reduction target, the target PTN) by ±10%. The results are shown in Table S3.

## Additional Information

**How to cite this article**: Liu, J. *et al*. Reducing human nitrogen use for food production. *Sci. Rep.* **6**, 30104; doi: 10.1038/srep30104 (2016).

## Supplementary Material

Supplementary Information

## Figures and Tables

**Table 1:** Comparisons of global nitrogen accounting for food.

Papers	Accounting for production, consumption or trade?	Food commodities or land use	Spatial scale	Spatial resolution	Temporal Period	Main focus
Smil^27^	Production	Crops and forages	Global	World	Mid-1990 s	N sources, sinks, and balance
Galloway *et al*.^24^	Production	Cropland	Global	World and Continents	1860, early 1990 s, 2050	Reactive N creation and budgets
Bouwman *et al*.^2^	Production	Cropland and grassland	Global	30 arc-min	1970–2030	Global and regional N balance
Galloway *et al*.^6^	Trade of fertilizer and grain	N.A.	Global	World	1860, 1995, 2005, 2050	Global N cycle
Bouwman *et al*.^2^	Production	Cropland and grassland	Global	30 arc-min	1970–2030	Soil N balance
Liu *et al*.^3^	Production	Cropland	Global	5 arc-min	2000	Nitrogen inputs, outputs and balance
Bouwman *et al*.^28^	Production	Arable land and grassland	Global	30 arc-min	1900–2050	Soil N budgets and fate
Bodirsky *et al*.^31^	Consumption	Crop and livestock products	Global	10 world regions	2010, 2050	N sources and losses
Erisman *et al*.^30^	Production	Crop and livestock products	Global	Global	1908–2010, 2010–2100	N fertilizer
This study	- Production - Consumption - Trade	- Vegetable commodities from cropland- Animal products commodities generated from cropland and pasture	Global	5 arc-min, national	1961–2000 2000–2030	Nitrogen footprint- Diagnostic for present-day using data- Embedded TN in trade of commodities 4 nitrogen oriented future scenarios
