# Supplementary material for: Reducing human nitrogen use for food production
Source: Sci Rep. 2016 Jul 22;6:30104. doi: 10.1038/srep30104 (PMC4957089; doi:10.1038/srep30104)
Supplement: Supplementary Information [file srep30104-s1.pdf]

Supplementary Information

Reducing human nitrogen use for food production

Junguo Liu[1*], Kun Ma[2], Philippe Ciais[3], Stephen Polasky[4]

[1]School of Environmental Science and Engineering, South University of Science and Technology of China, Shenzhen, 518055, China

[2]School of Nature Conservation, Beijing Forestry University, Qinghua East Road 35, Haidian District, 100083, Beijing, China; Tel.: +86-10-6233-6761

[3]Laboratoire des Sciences du Climat et de l'Environnement, CEA-CNRS-UVSQ, F-91191, Gif sur Yvette, France; Tel.: +33-16-908-9506

[4]Department of Applied Economics, University of Minnesota, St. Paul, Minnesota, United States; Tel.: +1-612-625-9213

*Corresponding author. Correspondence and requests for materials should be addressed to J.L. (E-mail: junguo.liu@gmail.com, water21water@yahoo.com, Tel.: +86-10-6233-6761, Fax: +86-10-6233-6761)

Author Contributions

J.L. developed the conceptual framework; J.L. and K.M. collected the data, performed the calculations, and created all figures; J.L., K.M., P.C, and S.P. discussed the results and wrote the paper.

Additional information

Supplementary information accompanies this paper at http://www.nature.com/

Competing financial interests: The authors declare no competing financial interests.

Supplementary Information – Figures

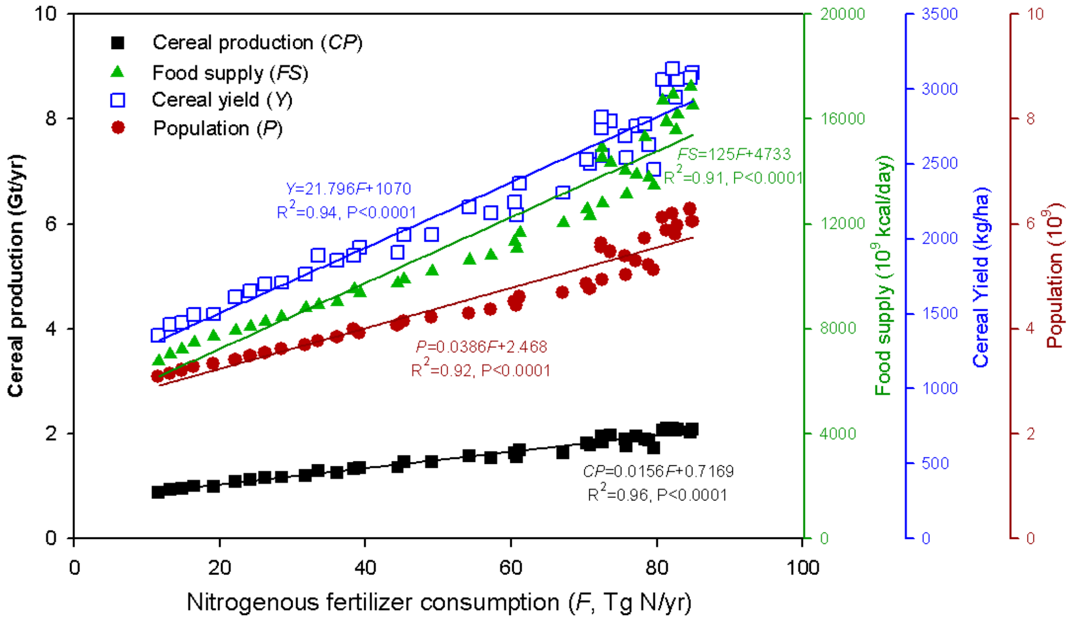

Fig. S1. Relationship between nitrogen fertilizer consumption and the food supply, cereal production, cereal yield, and population. Cereal production (*CP*) and food supply (*FS*) were both strongly linearly related to nitrogenous fertilizer consumption (*F*). This indicates that higher use of fertilizer contributes significantly to meeting the growing demand for food by the world's increasing population (*P*). Crop yield (*Y*) was also strongly linearly related to *F*, suggesting the importance of nitrogenous fertilizer for agricultural intensification. The data were obtained from the FAO[1]. Demand for cereals is projected to reach 2.864 to 3.229 Gt in 2050[2,3]. Based on the relationship between cereal production and fertilizer consumption, 138 to 161 Mt of nitrogenous fertilizer will be required to sustain production. This is an increase of 57 to 80 Mt compared with the 2000 level, and represents a 70 to 99% increase in the total consumption of nitrogenous fertilizer. The data are obtained from FAOSTAT[1].

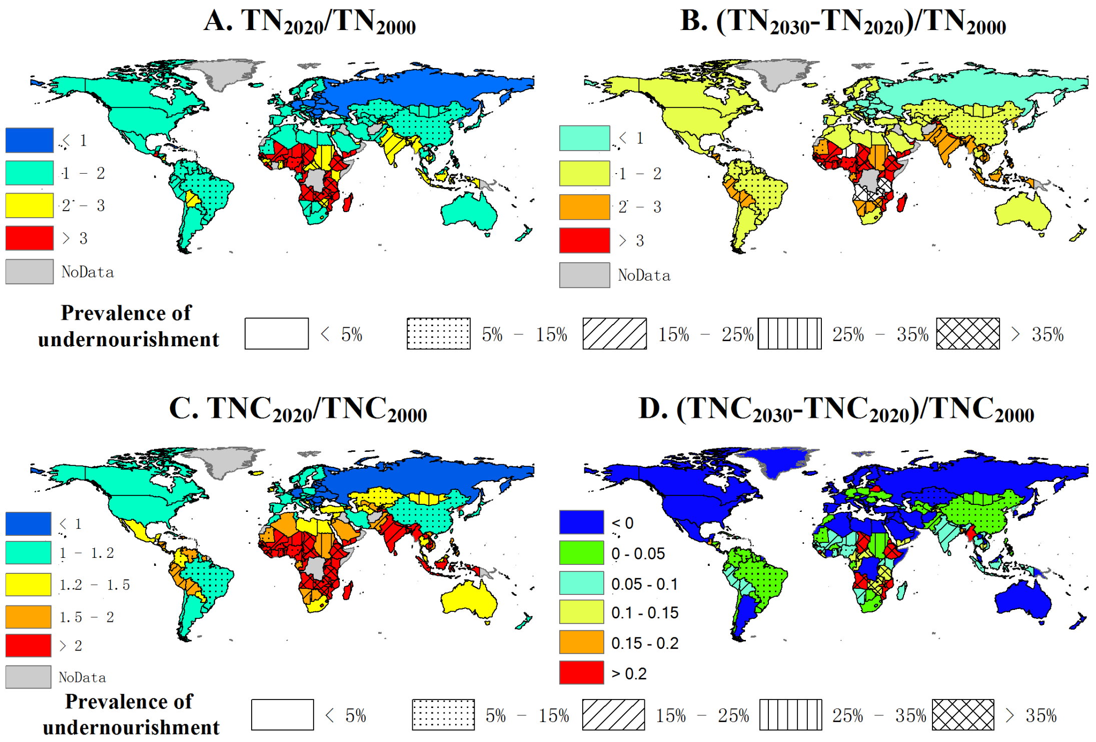

Fig. S2. Total nitrogen input (TN) of food production to meet the hunger eradication target (in the *baseline* scenario), and the relationship with the prevalence of undernourishment. TN_{2000}, TN_{2020}, and TN_{2030} are the TN in 2000, 2020, and 2030, respectively. TNC_{2000}, TNC_{2020}, and TNC_{2030} are the total nitrogen per capita (TNC) in 2000, 2020, and 2030, respectively. [*Created with ArcGIS 9.3.1*]

Supplementary Information

The lists of LIFD and non-LIFD countries.

Low-income food-deficit (LIFD) countries: Angola, Armenia, Azerbaijan, Bangladesh, Benin, Burkina Faso, Burundi, Cambodia, Cameroon, Central Africa, Chad, China, Comoros, Congo, Côte d'Ivoire, Democratic People's Republic of Korea (DPRK), Djibouti, Egypt, Eritrea, Ethiopia, Gambia, Georgia, Ghana, Guinea, Guinea-Bissau, Haiti, Honduras, India, Indonesia, Kenya, Kiribati, Kyrgyzstan, Laos, Lesotho, Liberia, Madagascar, Malawi, Mali, Mauritania, Moldova, Mongolia, Morocco, Mozambique, Nepal, Nicaragua, Niger, Nigeria, Pakistan, the Philippines, Rwanda, São Tome and Principe, Senegal, Sierra Leone, the Solomon Islands, Sri Lanka, Sudan, Swaziland, Syria, Tajikistan, Tanzania, Timor-Leste, Togo, Turkmenistan, Uganda, Uzbekistan, Vanuatu, Yemen, Zambia, Zimbabwe

Non-LIFD countries: Albania, Algeria, Antigua and Barbuda, Argentina, Australia, Austria, the Bahamas, Barbados, Belarus, Belgium, Belize, Bermuda, Bolivia, Bosnia and Herzegovina, Botswana, Brazil, Brunei Darussalam, Bulgaria, Canada, Cape Verde, Chile, Colombia, Costa Rica, Croatia, Cuba, Cyprus, the Czech Republic, Denmark, Dominica, the Dominican Republic, Ecuador, El Salvador, Estonia, Fiji, Finland, France, French Polynesia, Gabon, Germany, Greece, Grenada, Guatemala, Guyana, Hungary, Iceland, Iran, Ireland, Israel, Italy, Jamaica, Japan, Jordan, Kazakhstan, Kuwait, Latvia, Lebanon, Libya, Lithuania, Luxembourg, Malaysia, the Maldives, Malta, Mauritius, Mexico, Myanmar, Namibia, the Netherlands, the Netherlands Antilles, New Caledonia, New Zealand, Norway, the Occupied Palestinian Territory, Panama, Paraguay, Peru, Poland, Portugal, the Republic of Korea, Romania, Russia, Saint Kitts and Nevis, Saint Lucia, Saint Vincent, Samoa, Saudi Arabia, Serbia, the Seychelles, Slovakia, Slovenia, South

Africa, Spain, Suriname, Sweden, Switzerland, Thailand, the former Yugoslav Republic of Macedonia, Trinidad and Tobago, Tunisia, Turkey, Ukraine, United Arab Emirates, United Kingdom, United States, Uruguay, Venezuela, Vietnam.

Supplementary Information – Tables

Table S1. Total nitrogen input (TN) flows in net imports and net exports: amounts and national distribution.

**Country**	**Total TN in Net Imports (Tg yr^{-1})**	**Number of Countries that are Net Importer**	**Total TN in Net Exports (Tg yr^{-1})**	**Number of Countries that are Net Exporter**
Nitrogen-scarce countries (per capita NF< 9 kg N yr^{-1})	**12.49**	**85** [See **Group A** for a list of all countries in this category]	**0.05**	**9** [See **Group B** for a list of all countries in this category]
Nitrogen stressed countries (9 kg N yr^{-1}≤ per capita NF<15 kg N yr^{-1})	**7.60**	**27** [See **Group C** for a list of all countries in this category]	**1.97**	**23** [See **Group D** for a list of all countries in this category]
Other countries	**0.01**	**1** (*Niger*)	**11.26**	**8** [See **Group E** for a list of all

combined				countries in this category]
All the countries	**20.10**	**103**	**13.28**	**40**

Group A:

Albania, Algeria, Angola, Antigua and Barbuda, Armenia, Azerbaijan, Barbados, Bosnia and Herzegovina, Botswana, Brunei Darussalam, Burkina Faso, Burundi, Cambodia, Cameroon, Chile, Colombia, Commonwealth of Dominica, Congo, Côte d'Ivoire, Cyprus, Democratic People’s Republic of Korea, Dominican Republic, Egypt, Eritrea, Ethiopia, Gabon, Gambia, Georgia, Ghana, Guinea, Guinea-Bissau, Haiti, Indonesia, Israel, Italy, Jamaica, Japan, Jordan, Kenya, Kuwait, Kyrgyzstan, Laos, Latvia, Lebanon, Lesotho, Liberia, Libyan Arab Jamahiriya, Luxembourg, Madagascar, Malawi, Malaysia, Mali, Malta, Grenada, Mauritania, Mongolia, Morocco, Mozambique, Namibia, Nepal, the Netherlands, New Zealand, Nigeria, Norway, Panama, Peru, the Philippines, Portugal, Republic of Korea, Rwanda, Saint Kitts and Nevis, Saudi Arabia, Sierra Leone, South Africa, Sri Lanka, Swaziland, Switzerland, Tajikistan, Trinidad and Tobago, Tunisia, United Arab Emirates, Venezuela, Yemen, Zaire, Zambia

Group B:

Austria, Benin, Central African Republic, Nicaragua, Russian Federation, Suriname, Togo, Uganda, Vanuatu

Group C:

Bangladesh, Belgium, Bolivia, China, Costa Rica, Croatia, Cuba, Ecuador, El Salvador, Estonia, Finland, Germany, Greece, Guatemala, Honduras, Iran, Ireland, Mexico, Pakistan, Poland, Senegal, Slovenia, Spain, Syrian Arab Republic, Turkey, the United Kingdom, Zimbabwe

Group D:

Belize, Bulgaria, Chad, Czech Republic, France, Guyana, Hungary, India, Kazakhstan, Lithuania, Moldova, Myanmar, Romania, Saint Vincent and the Grenadines, Slovakia, Sudan, Sweden, Thailand, Turkmenistan, Ukraine, Uruguay, Uzbekistan, Vietnam

Group E:

Argentina, Australia, Brazil, Canada, Denmark, Paraguay, Saint Lucia, the United States

Table S2. Estimated amount of nitrogen required per calorie of food in different components of the human diet.

This table shows the world averages and the ranges based on results from different continents.

**Food type**	**Product TN (g N/kg food)**	**N required per unit food energy (g N/1000 kcal)**
Cereals	39.6 (28.8–45.9)	14.4 (10.7-17.3)
Starchy roots	7.8 (4.2-10.3)	10.3 (4.4-14.6)
Sugar crops	3.4 (2.7-4.0)	8.4 (2.8-12.9)
Pulses	147.1 (55.2-183.9)	43.4 (17.4-53.7)
Oil crops	67.9 (50.3-78.7)	21.8 (10.4-30.3)
Plant products	26.5 (14.9-32.3)	15.7 (9.9-18.6)
Animal products	—	83.9 (47.3-102.3)

Table S3. The uncertainty ranges of global TN in different scenarios.

Region	DIET (S1)	WASTE (S2)	Efficiency (S3)	Hybrid (S4)
2020-LIDF	142–170	128–152	108–145	97–131
2020-NON-LIDF	74–90	84–90	71–87	51–70
2020-Global	216–260	212–242	178–232	148–201
2030-LIDF	161–197	142–165	105–155	91–131
2030-NON-LIDF	78–95	83–87	63–84	43–62
2030-Global	239–292	225–252	169–239	134–194

References

1 FAO. *FAOSTAT: FAO statistical databases*. (2014). Available at: http://faostat3.fao.org/home/index.html. Date of access: 15/10/2014.
